# Moderators of treatment effect in a randomised controlled trial of single‐ and multi‐family therapy for anorexia nervosa in adolescents and emerging adults

**DOI:** 10.1002/erv.3050

**Published:** 2023-11-27

**Authors:** Julian Baudinet, John Hodsoll, Ulrike Schmidt, Mima Simic, Sabine Landau, Ivan Eisler

**Affiliations:** ^1^ Centre for Research in Eating and Weight Disorders Institute of Psychiatry, Psychology and Neuroscience King's College London London UK; ^2^ Maudsley Centre for Child and Adolescent Eating Disorders South London and Maudsley NHS Foundation Trust London UK; ^3^ Department of Biostatistics and Health Informatics School of Mental Health and Psychological Sciences Institute of Psychiatry, Psychology and Neuroscience King's College London London UK; ^4^ Adult Eating Disorders Service South London and Maudsley NHS Foundation Trust London UK

**Keywords:** adolescents, anorexia nervosa, emerging adults, family therapy, family‐based treatment, group, Maudsley family therapy, multi‐family therapy, randomised controlled trial, transition age youth

## Abstract

**Introduction:**

Multi‐family therapy for anorexia nervosa (MFT‐AN) is a novel, group‐based intervention that intensifies single‐family therapy for anorexia nervosa (FT‐AN), with the aim of improving outcomes. The current study explored treatment moderators in a randomised controlled trial (*N* = 167) of FT‐AN and MFT‐AN for young people (adolescents/emerging adults aged 13–20 years) with anorexia nervosa.

**Methods:**

Data were analysed using multiple linear regression. Six hypothesised baseline participant and parent factors were tested as possible moderators of treatment effect on end‐of‐treatment and follow‐up percentage of median Body Mass Index (%mBMI); age, eating disorder symptom severity, perceived family conflict (young person and parent ratings) and parent‐rated experiences of caregiving (positive and negative).

**Results:**

Greater parent‐rated positive caregiving experiences moderated treatment outcomes at follow‐up (*β* = −0.47, 95%CI: −0.91, −0.03, *p* = 0.04), but not end‐of‐treatment. Participants who had fewer parent‐rated positive caregiving experiences at baseline had higher weight at follow‐up if they had MFT‐AN compared to FT‐AN. No other hypothesised baseline factors moderated treatment outcome (*p*'s > 0.05).

**Discussion:**

The current study suggests MFT‐AN may be indicated for families who present with fewer positive caregiving experiences to treatment. The MFT‐AN group context may help to promote mentalisation and hope for these families, which may be harder to achieve in single‐family treatment. Future research is needed to empirically evaluate how and why MFT‐AN supports this group more.

**Trial Registration:**

ISRCTN registry: ISRCTN11275465, registered 29 January 2007.

## Highlights

- The current study suggests multi‐family therapy for anorexia nervosa (MFT‐AN) may be indicated for families who present to treatment with fewer positive caregiving experiences.
- The MFT‐AN group context may help to promote mentalisation and hope for these families, which may be harder to achieve in single‐family treatment.
- This study did not find evidence that age, eating disorder symptom severity, perceived family conflict or level of negative caregiving experiences moderated treatment effect.

## 1 INTRODUCTION

In recent decades, there has been a significant increase in the development and evaluation of eating disorder treatments for young people. Eating disorder focused family therapy is consistently recommended in international guidelines as the first line recommended treatment for children and adolescents (Hilbert et al., 2017; Monteleone et al., 2022) and promising findings are emerging for young adults (also referred to as transition age youth) (Dimitropoulos et al., 2018; Nyman‐Carlsson et al., 2020). While family treatments are effective for most at reducing eating disorder psychopathology and supporting weight restoration for those who are underweight (Fisher et al., 2019; Jewell et al., 2016), current treatments are by no means sufficient for all and many require additional and/or alternative interventions.

Family interventions, by definition, include parental/caregiver involvement in treatment. Typically, parents are encouraged to take a lead on supporting their child to manage eating disorder symptoms when they are struggling to do so themselves (Eisler, Simic, Blessitt, et al., 2016). Given that parenting practices change quite considerably depending on a child's age and development stage, the role that parents take in a young person's treatment may vary considerably (Dodge et al., in press). Questions regarding the suitability of family treatment for older or more independent young people is often debated (Dimitropoulos et al., 2015, 2016). This is especially true for emerging adults (age 18–25 years), who are in a unique developmental period marked by multiple life transitions, an increasing societal expectation to be independent (at least in western cultures), but often with more parental involvement than might be expected of adults aged in their late 20s and higher (Potterton et al., 2020).

To better understand for whom family therapy interventions are most and least effective, the field has increasingly turned to the empirical investigation of the process of change during treatments. Recent reviews of specific moderators of treatment effect, as well as non‐specific predictors of outcome, indicate early change and several baseline young person and family factors may impact treatment response (Gorrell et al., 2022; Hamadi & Holliday, 2020; Vall & Wade, 2015).

Specifically, early weight gain (Doyle et al., 2010; Le Grange et al., 2014; Madden et al., 2015), and lower baseline eating disorder symptom strength and level of eating disorder obsessionality (Le Grange et al., 2012) have been associated with improved end‐of‐treatment outcomes. Additionally, family factors, such as parental mentalising capacity (Jewell et al., 2021), and parental self‐efficacy (Byrne et al., 2015; Le Grange et al., 2012) have also been shown to influence treatment response. Lastly, therapeutic alliance may also impact outcomes (Forsberg et al., 2014; Graves et al., 2017; Jewell et al., 2021).

Several novel family‐focussed interventions have been developed in recent years to provide additional supports and increase treatment intensity to those who may need more than outpatient treatment typically provides (Richards et al., 2018). Many of these novel treatments target the aforementioned factors associated with poorer treatment response as well as the bio‐psychosocial factors associated with eating disorders. Intensifications of family therapy model have included brief individualised intensive‐outpatient treatments (Fink et al., 2017; Rockwell et al., 2011), family‐informed day‐patient services (Baudinet et al., 2020; Baudinet & Simic, 2021; Hoste, 2015; Simic et al., 2018), home treatment (Herpertz‐Dahlmann et al., 2021), and intensive multi‐family group‐based interventions (Baudinet, Eisler, Dawson, et al., 2021).

Multi‐family therapy models have now been developed for both anorexia nervosa (MFT‐AN; Simic et al., 2021; Tantillo et al., 2020) and bulimia nervosa (MFT‐BN; Escoffié et al., 2022; Stewart et al., 2019). The unique group‐based MFT context and content aims to reduce isolation, promote solidarity and increase treatment intensity (Dawson et al., 2018; Simic & Eisler, 2015). Emerging evidence indicates MFT is acceptable to adolescents and young adults and is associated with a range of physical, psychological and well‐being improvements (Baudinet, Eisler, Dawson, et al., 2021).

Outcomes from the only outpatient randomised control trial (RCT) of MFT‐AN indicate that the addition of MFT‐AN to single‐family therapy for anorexia nervosa (FT‐AN) is associated with improved outcomes compared to FT‐AN alone (Eisler, Simic, Hodsoll, et al., 2016). However, little is known about the moderators of treatment effect. Qualitative data on the perceived change mechanisms show that young people, parents and clinicians generally agree on how change is perceived to occur in MFT‐AN (Baudinet et al., 2023; Baudinet et al., in press; Wiseman et al., 2019). Specifically, the power and intensity of the group‐context is thought to help disrupt difficult and stuck family/team dynamics, which can increase hope, motivation and mentalisation capacity through mechanisms of increased support, reduced isolation and comparisons with others with similar difficulties (Baudinet et al., 2023; Baudinet et al., in press).

Given the unique group‐based setting, the additional intensity and support available, and new sources of learning, MFT‐AN could be well placed to target several of the identified moderators, mediators and predictors of treatment response (Gorrell et al., 2022; Hamadi & Holliday, 2020). Specifically, It has been suggested that (a) the increased intensity and additional support with mealtimes may help those with greater illness severity, (b) the peer support, broad therapeutic content and new channels of learning (e.g. via activities, from peers, with practical support at mealtimes) may help families who are feeling demoralised, hopeless or struggling with conflict and (c) the mechanisms for greater young‐person only treatment content may mean MFT‐AN is more beneficial for older adolescents. Nevertheless, no research to date has investigated this.

The current study aims to confirm the moderating effect of six hypothesised moderators on weight at the end of treatment and follow‐up in the Eisler, Simic, Hodsoll, et al. (2016, 2016) RCT. The baseline moderators of interest are 1) age, 2) eating disorder illness severity, 3) perceived family conflict and 4) parent‐rated experience of caregiving. Specifically, it was hypothesised that MFT‐AN would be more effective than FT‐AN for older adolescents/emerging adults, with more severe symptoms and family difficulties due to the increased intensity and support offered early in treatment and the potentially more developmentally appropriate and flexible nature of the MFT‐AN context for older adolescents.

## 2 METHOD

### 2.1 Study design and ethical approval

Data from a pragmatic multicentre RCT that compared outcomes of FT‐AN and MFT‐AN (Eisler, Simic, Hodsoll, et al., 2016) were used in this study. Both treatments lasted 12 months and assessment points during the trial included baseline, 3 months (early change), 12 months (end of treatment) and 18 months (6‐month follow‐up). The trial was registered with Current Controlled Trials (registration number ISRCTN11275465) and with the UK Clinical Research Network (UKCRN ID 6041). Ethical approval for the study was given by the UK Integrated Research Approval System Committee (04/MREC/022). All participants provided informed consent. Further details of this design, details of block randomisation procedure and main outcome findings are reported elsewhere (Eisler, Simic, Hodsoll, et al., 2016).

### 2.2 Participants

Participants were 167 adolescents and their family members; 82 were randomly allocated to FT‐AN and 85 to MFT‐AN. To be eligible for the RCT participants needed to be (a) aged between 13 and 20 years, (b) fulfil diagnostic and statistical manual (fourth edition; DSM‐IV) (American Psychiatric Association, 1994) criteria for anorexia nervosa or eating disorder not otherwise specified (EDNOS, restricting type), (c) and either be below 86% median Body Mass Index (%mBMI) for age and sex or have lost 15% body weight in the last 3 months. Eating disorder not otherwise specified restrictive subtype included young people who met some, but not all, of the criteria for anorexia nervosa and experienced clinically significant levels of distress and/or impairment. This included, but is not limited to, not meeting the low weight or amenorrhoea criterion. Exclusion criteria included a learning disability, psychosis, living in care, alcohol or substance dependence, coexisting medical condition(s) that might impact weight, being at serious medical risk due to extremely low body weight (<67%mBMI) or medical instability. Medical instability was defined as experiencing one or more of the following: dehydration, (a postural drop of 20 mmHg), bradycardia (pulse rate below 40 beats per minute), failed squat test (unable to get up without using arms as levers), temperature below 34.5°C or electrolyte imbalance.

### 2.3 Treatments

#### 2.3.1 Family therapy for anorexia nervosa

Family therapy for anorexia nervosa (FT‐AN) is a specific, manualised, evidence‐based, four phase treatment for adolescent anorexia nervosa (Eisler, Simic, Blessitt, et al., 2016). Over the course of 12 months, young people and their families are initially seen weekly, which becomes less frequent as treatment progresses. Treatment initially focuses on engagement and development of the therapeutic alliance (phase 1) and supporting the family to manage the eating disorder symptoms (phase 2). Once the young person is able to eat more independently and is more stable medically, treatment shifts to developmental and family lifecycle needs (phase 3). The final phase of treatment (phase 4) focuses on endings and discharge from treatment. Sessions are mainly conjoint family meetings although some individual sessions are included where appropriate (particularly with older adolescents) (cf Baudinet, Simic, & Eisler, 2021). See original outcomes paper for further details (Eisler, Simic, Hodsoll, et al., 2016).

#### 2.3.2 Multi‐family therapy for anorexia nervosa

Multi‐family therapy for anorexia nervosa (MFT‐AN) (Simic et al., 2021) is based on the same theoretical principles as FT‐AN but provides a more intensive group‐based format for treatment. It also extends the model by delivering treatment content via verbal and non‐verbal activities, role plays, repeated practical mealtime support, and group‐based sharing of strengths and concerns with people in a similar position; all with the support of a clinical team. MFT‐AN starts with an intensive 4‐day multi‐family group intervention for 5–8 families. This is followed by an additional six 1‐day meetings at 2–8 week intervals spread over 9 months. Individual family meetings are provided in between MFT‐AN days as needed. The total duration of treatment was 12 months. See the treatment manual (Simic et al., 2021) and main outcome paper (Eisler, Simic, Hodsoll, et al., 2016) for further details.

### 2.4 Assessment and outcome metrics

#### 2.4.1 Baseline variables

The following six variables were assessed as potential moderators of treatment effect on outcome. Two of the variables have 2 subscales giving a total of 6 moderators. Only the family functioning measure is described in detail below as all other measures are described in the primary outcome paper (Eisler, Simic, Hodsoll, et al., 2016).

1. Age (years)
2. Eating disorder psychopathology (Eating Disorder Examination Questionnaire (EDE‐Q) global score) (Fairburn & Cooper, 1993). It has been shown to have moderate to good internal consistency (Cronbach's alpha 0.73–93) and high test‐retest reliability (*r* > 0.89) (Rose et al., 2013).
3. Perceived family conflict was assessed using the conflict subscale of the Self‐Report Family Inventory (SFI; Beavers & Hampson, 1993). This has self‐reports from the young person and caregiver perspectives giving two potential moderators. The SFI is a 36‐item self‐report measure of family functioning with five scales: (a) health/competence (perceived family affect, parental coalitions, problem‐solving abilities, autonomy and individuality, optimistic vs. pessimistic views, and acceptance of family members), (b) conflict (perceived overt vs. covert conflict, including arguing, blaming, fighting openly, acceptance of personal responsibility, unresolved conflict, and negative feeling tone), (c) cohesion (perceived family togetherness, satisfaction received from inside the family vs. outside, and spending time together), (d) leadership (perceived parental leadership, directiveness, and degree of rigidity of control), and (e) emotional expressiveness (perceived verbal and nonverbal expression of warmth, caring and closeness). All items are rated on five‐point Likert scales with lower scores on all subscales indicative of improved functioning. The SFI has demonstrated good internal consistency (Cronbach alpha 0.84–0.93) and good test‐retest reliability (*r* > 0.85) (Beavers & Hampson, 2000).
4. Perceived caregiving burden was measured using the Experience of Caregiving Inventory (ECI; Szmukler et al., 1996). The ECI has 2 subscales (positive and negative) for which moderation was examined separately. The ECI was assessed as ‘excellent’ on the Consensus‐based Standards for the selection of health Measurement Instruments criteria (COSMIN; Mokkink et al., 2010) in a recent review (Hilton et al., 2022). It has also been shown to have moderate to good internal consistency (Cronbach's alpha 0.74–0.91) (Szmukler et al., 1996).

#### 2.4.2 Outcome variables

The primary outcome in this study was the percentage of median Body Mass Index (%mBMI) at 6‐month follow‐up (T4; 18 months post randomisation), as this was the timepoint for which treatment effect was significant in the primary outcome paper (Eisler, Simic, Hodsoll, et al., 2016, 2016). The secondary outcome investigated was end of treatment %mBMI (T3; 12 months post randomisation).

All participants were assessed as requiring weight gain. Two participants had a weight higher than 90%mBMI at assessment. Both were female sex assigned at birth and not menstruating regularly. All young people above 85%mBMI were either premenarchal (*n* = 2), were experiencing secondary amenorrhoea (*n* = 11) or had irregular periods (*n* = 6). Eating disorder illness severity (EDE‐Q global scores) were initially also considered as an outcome variable, however, was not included due to level of missing data at T3 and T4.

### 2.5 Statistical analyses

A set of six linear mixed models were used to determine whether each of the identified baseline variables (age, EDE‐Q global score, young person SFI conflict score, parent/caregiver SFI conflict score, parent/caregiver positive experiences of caregiving and negative experiences of caregiving), moderated treatment effect on the primary outcome (%mBMI). Fixed effects were treatment type (FT‐AN vs. MFT‐AN), time (T2, T3 and T4 or 3, 12 and 18 months post randomisation), baseline %mBMI, two‐way interactions between treatment type and time, the moderator and time, treatment type and the moderator, and the 3‐way interaction between treatment, time and the baseline moderator. A subject‐varying random intercept was included to account for the dependencies between repeated measures. Linear contrasts for the two‐way interaction between treatment and the baseline variable estimated moderation at T3 and T4, with 95% confidence intervals to give the range of plausible values for the estimate.

There was some attrition for the primary outcome at both end of treatment (19 values, 11.4% missing) and 6 months post treatment (52 values, 31.1% missing). As described in Eisler, Simic, Hodsoll, et al. (2016, 2016), several baseline variables predicted missingness (family history of eating disorder, co‐morbidity [binary yes/no variable] and birth order in family), as well as not completing the full 12 months of treatment. There was also some missing data for baseline variables (range 0–42; median 38.5 values, 23.1% missing). Multiple imputation (MI, with 100 imputations) was used to generate inferences valid under a missing at random assumption. To ensure any potential treatment group by baseline variable moderation effects were not diluted in the imputation process, one imputation model was generated by treatment group (including the baseline variables). Baseline variables were centred for use in the interaction terms for ease of interpretation and to deal with potential multi‐collinearity (variables were centred by dataset after imputation). Linear mixed models were fit for each imputed data set and statistical estimates pooled across the 100 datasets according to Rubin's rules. Statistical significance was set to *p* < 0.05. Given there are 12 contrasts of interest, this increases the risk of a false positive. However, given this is the only trial comparing FT‐AN and MFT‐AN we wish to guard against being unable to identify potential avenues for further research and present the results with a view to hypothesis generation.

Level 1 and level 2 model residuals were inspected from the first imputed dataset to ensure they met the required assumptions of linear mixed models (visual inspection of histograms, scatterplots and P‐P plots of residuals indicate the of normality of errors and homoscedasticity of the data). STATA release 17 software (StataCorp, 2021) was used to conduct these analyses and R 4.2 using the ggplot2 package (Wickham, 2016) to generate figures.

## 3 RESULTS

### 3.1 Sample characteristics

Given a detailed description of baseline participant characteristics have been published elsewhere (Eisler, Simic, Hodsoll, et al., 2016), only a summary is provided here. Mean age at baseline was 15.7 years (sd = 1.65), 91.0% were female and 90.4% self‐identified their ethnicity as White. Mean weight (%mBMI) at baseline was 80.0% (sd = 6.06). Three quarters (76.1%) met diagnostic criteria for anorexia nervosa with the remaining classified as EDNOS (restricting type). See Table 1 for further details.

**TABLE 1:** Demographics and clinical variables at baseline.

	FT‐AN							MFT‐AN						
	N	Missing		Mean	sd	Min.	Max	N	Missing		Mean	sd	Min.	Max.
		n	%						n	%				
Young person baseline (T1) factors														
Age	82	0	0.0%	15.65	1.60	12.95	20.10	85	0	0.0%	15.71	1.71	12.75	20.88
Weight (%mBMI)	82	0	0.0%	78.36	5.83	67.60	96.80	85	0	0.0%	77.57	6.28	65.05	91.90
Duration of illness (months)	76	6	7.3%	11.39	12.56	1	60	73	12	14.1%	9.64	10.53	0	55
EDE‐Q global score	62	20	24.4%	3.27	1.50	0.00	5.70	68	17	20.0%	3.19	1.68	0.00	5.69
SFI‐conflict	61	21	25.6%	24.13	8.15	12	44	64	21	24.7%	23.85	9.13	11	51
Parent baseline (T1) factors														
SFI‐conflict	63	19	23.2%	22.25	7.90	11	44	68	17	20.0%	21.59	6.61	11	38
ECI‐negative	61	21	25.6%	86.28	28.40	27	152	65	20	23.5%	84.88	32.16	21	157
ECI‐positive	61	21	25.6%	28.84	7.86	11	45	66	19	22.4%	27.74	9.22	9	48

Abbreviations: %mBMI, percentage of median Body Mass Index; ECI, Experience of Caregiving Inventory; EDE‐Q, Eating Disorder Examination Questionnaire; FT‐AN, family therapy for anorexia nervosa; MFT‐AN, multi‐family therapy; SFI, Self‐Report Family Inventory.

### 3.2 Moderators of outcome

#### 3.2.1 End‐of‐treatment

None of the variables assessed were significant moderators of treatment effect at end of treatment (all *p*'s > 0.05). See Table 2.

**TABLE 2:** Moderator analysis of interaction between baseline variables and treatment type (Multi‐family therapy for anorexia nervosa (MFT‐AN) vs. Family therapy models for anorexia nervosa (FT‐AN) as reference group) on weight (%mBMI) at end of treatment (T3) and follow‐up (T4).

	End‐of‐treatment (T3) %mBMI				Follow‐up (T4) %mBMI			
	95%CI				95%CI			
	*β*	Lower	Upper	*p*	*β*	Lower	Upper	*p*
Baseline (T1) factors								
Age	1.16	−0.60	2.92	0.20	1.30	−0.56	3.16	0.17
EDE‐Q global	−0.48	−2.50	1.55	0.65	−0.08	−2.24	2.08	0.94
SFI‐conflict								
Young person	−0.02	−0.45	0.41	0.94	0.05	−0.44	0.55	0.84
Parent	0.27	−0.17	0.72	0.23	0.14	−0.33	0.60	0.56
Experience of caregiving								
ECI‐negative	0.03	−0.08	0.15	0.58	0.01	−0.11	0.14	0.84
ECI‐positive	−0.27	−0.76	0.23	0.29	−0.47	−0.91	−0.03	0.04

Abbreviations: %mBMI, percentage of median Body Mass Index; ECI, Experience of Caregiving Inventory; EDE‐Q, Eating Disorder Examination Questionnaire; SFI, Self‐Report Family Inventory.

#### 3.2.2 Follow‐up

**FIGURE 1:**
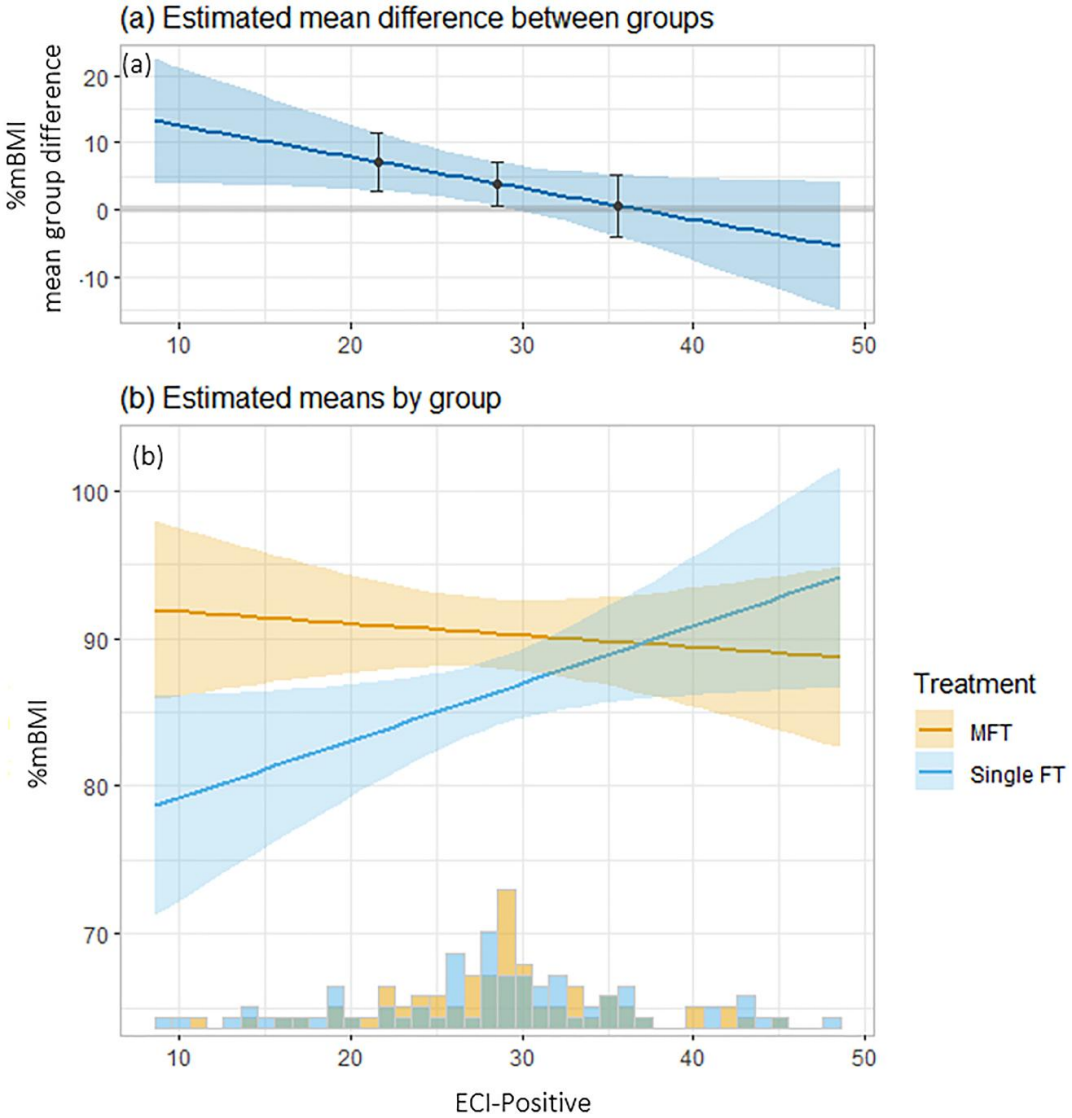
Moderating effect of baseline parent rated positive caregiving experiences (ECI‐positive) on weight (%mBMI) at follow‐up (T4). Figure (a) shows how the predicted mean difference in %mBMI between treatment groups at T4 changes with the baseline value of ECI‐positive ratings. Contrasts are shown at the mean ± 1 standard deviation of the Experience of Caregiving Inventory (ECI). (b) shows how the predicted mean %mBMI at T4 changes by baseline ECI (positive) together with a histogram showing the distribution of raw baseline ECI positive scores by treatment group. %mBMI, percentage of median Body Mass Index; ECI‐positive, positive subscale of the Experience of Caregiving Inventory; FT, family therapy; MFT, multi‐family therapy; sd, standard deviation. Error ribbons represent 95% confidence intervals.

Baseline positive caregiving experience (ECI‐positive) was the only significant treatment moderator at follow‐up %mBMI (*β* = −0.47, 95% CI: −0.91, −0.03, *p* = 0.04). Lower positive caregiving experience at baseline was associated with a poorer response (lower %mBMI) at follow‐up for those who received FT‐AN alone. In contrast, outcomes were similar regardless of parent‐rated baseline positive caregiving experiences for those who received MFT‐AN. See Figure 1.

Age, eating disorder symptom severity (EDE‐Q global score), perceived family conflict (SFI‐conflict) and parent‐rated negative experiences of caregiving (ECI‐negative) were not significant moderators of treatment effect at follow‐up (all *p*'s > 0.05).

## 4 DISCUSSION

The aim of this study was to explore whether six hypothesised baseline factors moderated treatment effect in a RCT of FT‐AN and MFT‐AN for young people with anorexia nervosa. The results identified one treatment moderator of outcome at follow‐up; caregiver‐rated baseline positive caregiver experiences. Specifically, families presenting to treatment with fewer parent‐rated positive caregiving experiences had lower weight at follow‐up in FT‐AN compared to MFT‐AN. Whereas, MFT‐AN seemed to protect against the impact of this, with follow‐up weight similar irrespective of perceived positive experiences at baseline. This study did not find evidence that age, eating disorder symptom severity, perceived family conflict or level of negative caregiving experiences moderated treatment effect.

The main finding, regarding parent‐rated positive caregiving experiences, fits with previous research from one study that maternal and paternal warmth, measured using the whole family interview‐based Standardized Clinical Family Interview (SCFI; Kinston & Loader, 1984), may be associated with improved outcomes in family treatment for anorexia nervosa (Le Grange et al., 2011). One possible explanation is that more parent‐rated positive caregiving experiences may be linked to greater parental hope and emotion regulation skills, in the sense that parents may be better able to use reappraisal techniques to regulate their emotions. Holding hope and noticing the positives, finding meaning or identifying a silver lining in a difficult situation are all adaptive ways of managing strong emotional states and difficult situations (McRae, 2016). For families struggling to notice the positives, the group‐based MFT‐AN context may help to promote this via increased mentalisation and peer‐support (Baudinet et al., 2023). Additionally, other unaccounted for factors may be associated with positive caregiving experiences, such as length of the young person's untreated eating disorder. Arguably, parents whose child has a shorter illness duration may be more able to see the positives, as opposed to parents of a young person who has been unwell for a long time. Further investigation is required to better understand this finding.

Contrary to our hypothesis, the current findings suggest the FT‐AN and MFT‐AN treatment formats may be equally effective for younger and older adolescents, including emerging adults up to 20 years of age. Emerging adulthood is a time of multiple life and service transitions, during which there is often a desire for and/or expectation of increasing independence (McClelland et al., 2020; Potterton et al., 2020; Treasure et al., 2005). While it was expected that the MFT‐AN context, with more opportunity for peer‐to‐peer and inter‐familial therapeutic engagement, may be better suited to older participants, the current data do not provide evidence for this assertion. This directly challenges some commonly held clinical impressions, for example, that FT‐AN may not meet the needs or be as effective for older adolescents who require more independence. However, it is also important to note that the absence of evidence is not evidence of absence and this may be due to a lack of power in the statistical analyses. Independent replication of these findings with an appropriate sample size are needed.

Additionally, the data do not support the hypothesis that MFT‐AN would be more effective for people with more severe eating disorder symptomatology. Previous research has reported that young people with higher levels of baseline eating disorder psychopathology may have better outcomes in conjoint family therapy as opposed to individual therapy (Le Grange et al., 2012) or parent‐only treatment (Le Grange et al., 2016). The current findings add to this literature by demonstrating that higher symptom severity can be managed similarly as effectively in either FT‐AN or MFT‐AN, although the greater resources needed for MFT‐AN should also be considered when determining treatment choice.

Interestingly, neither parent‐ nor young person‐perceived family conflict moderated treatment effect. Previous studies have reported that families with higher levels of criticism or hostility (a potentially related albeit different construct to conflict) may be associated with poorer outcomes, which is ameliorated by offering separated, as opposed to conjoint, family treatment (Eisler et al., 2007; Simic et al., 2020). Qualitative data on the experience of MFT suggests that the group‐context can help to increase hope, motivation and mentalisation capacity through mechanisms of increased support, reduced isolation and comparisons with others with similar difficulties (Baudinet et al., 2023; Escoffié et al., 2022). As such, it could be argued that the more flexible and public nature of MFT‐AN may have ameliorated against existing familial conflict. Nevertheless, the data do not support this and suggests high conflict families will have similar outcomes in either FT‐AN or MFT‐AN. Of course, this may simply be the result of issues with measurement, given perceived level of conflict is likely very subjective, varies depending on how it is measured, and from whose perspective it is assessed. Hopefully upcoming MFT‐AN RCTs (Baudinet, Eisler, Simic, et al., 2021; Carrot et al., 2019) can begin to answer some of these questions.

In many ways, the current pattern of results is surprising. On the whole they do not seem to match the available qualitative data on the experience of MFT‐AN or how change is perceived to occur form the young person, parent or clinician perspective (Baudinet, Eisler, Dawson, et al., 2021; Baudinet et al., in press; Baudinet et al., 2023), nor the current theoretical model of change. This may partly be due to FT‐AN and MFT‐AN being very similar treatments and theoretically derived from the same models. In many ways, the Eisler, Simic, hodsoll, et al. (2016, 2016) trial is a comparison of FT‐AN with/without the addition of adjunctive MFT‐AN. Perhaps the treatments were too similar for the hypothesised factors to moderate their effects. Unfortunately, too little is currently known of the process of change in either treatment for further comment. More research on the treatment mechanisms of both is needed.

### 4.1 Strengths and limitations

The sample size and RCT design are both strengths of the study and add confidence in interpretations of the current findings. Nevertheless, the analysis was exploratory and correction for multiple analyses not applied. This allowed for a wider exploration of the findings, however, concomitantly, increases the risk of Type I errors. Some moderator confidence intervals were also relatively wide (e.g., for age and EDE‐Q global score) indicating that greater precision may have been necessary (more statistical power) to detect some of the hypothesised interactions. Additionally, there was a relatively large amount of missing data for some variables. Multiple imputation procedures were employed to limit any biases due to missingness, however, these may not eliminate all such biases. Caution is therefore needed in how the findings from the study are interpreted and targeted replication is needed.

Lastly, the primary outcome variable in this study was %mBMI and eating disorder symptom change was not considered. Weight is only one component of recovery from anorexia nervosa and future research is needed to understand whether these, or other, factors moderate treatment effects on symptom change during treatment.

## 5 CONCLUSION

Current results extend previous findings by demonstrating that MFT‐AN may be associated with improved weight outcomes compared to single‐family therapy for families presenting to treatment with fewer baseline positive caregiving experiences. For these families, single‐family therapy may be counter‐indicated. Alternatively, MFT‐AN may act to buffer against the negative impact that low levels of positive caregiving may have on weight outcomes at follow‐up. Nevertheless, several hypothesised treatment moderators were not significant and highlight the lack of knowledge and empirical data evaluating the underlying mechanisms of change in either FT‐AN or MFT‐AN. More studies and replication of these findings are needed to confirm these conclusions.

## AUTHOR CONTRIBUTIONS

Julian Baudinet conceived of the current study, conducted the initial statistical analyses, and prepared the manuscript. John Hodsoll conducted the final statistical analyses, contributed to study design and manuscript preparation. Ulrike Schmidt and Ivan Eisler supervised the work. SL consulted on and supervised the statistical analyses. Mima Simic contributed to study design and manuscript preparation.

## CONFLICT OF INTEREST STATEMENT

Julian Baudinet, Ivan Eisler, and Mima Simic, all receive royalties from Routledge for a published manual for MFT‐AN (Simic et al., 2021).

## Data Availability

Data will be made available upon reasonable request for those participants who have consented to this.

## References

[erv3050-bib-0001] American Psychiatric Association . (1994). Diagnostic and statistical manual of mental disorders: DSM‐IV (4th ed.). American Psychological Association.

[erv3050-bib-0002] Baudinet, J. , Eisler, I. , Dawson, L. , Simic, M. , & Schmidt, U. (2021a). Multi‐family therapy for eating disorders: A systematic scoping review of the quantitative and qualitative findings. International Journal of Eating Disorders, 54(12), 2095–2120. 10.1002/EAT.23616 34672007 PMC9298280

[erv3050-bib-0003] Baudinet, J. , Eisler, I. , Konstantellou, A. , Hunt, T. , Kassamali, F. , McLaughlin, N. , Simic, M. , & Schmidt, U. (2023). Perceived change mechanisms in multi‐family therapy for anorexia nervosa: A qualitative follow‐up study of adolescent and parent experiences. European Eating Disorders Review, 31(6), 822–836. Embase Weekly Updates. 10.1002/erv.3006 37415392

[erv3050-bib-0004] Baudinet, J. , Eisler, I. , Roddy, M. , Turner, J. , Simic, M. , & Schmidt, U. Clinician perspectives on the change mechanisms in multi‐family therapy for anorexia nervosa: A qualitative study. Family Process. (in press). 10.1186/s40337-024-01064-2 PMC11267764 39049063

[erv3050-bib-0005] Baudinet, J. , Eisler, I. , Simic, M. , & Schmidt, U. (2021b). Brief early adolescent multi‐family therapy (BEAM) trial for anorexia nervosa: A feasibility randomized controlled trial protocol. Journal of Eating Disorders, 9(1), 71. 10.1186/s40337-021-00426-4 34134769 PMC8206871

[erv3050-bib-0006] Baudinet, J. , & Simic, M. (2021). Adolescent eating disorder day programme treatment models and outcomes: A systematic scoping review. Frontiers in Psychiatry, 12, 539. 10.3389/fpsyt.2021.652604 PMC8116630 33995149

[erv3050-bib-0064] Baudinet, J. , Simic, M. , & Eisler, I. (2021). From treatment models to manuals: Maudsley single‐ and multi‐family therapy for adolescent eating disorders. In M. Mariotti , G. Saba , & P. Stratton (Eds.), Systemic approaches to manuals (1st ed., pp. 349–372). Springer, Cham. 10.1007/978-3-030-73640-8

[erv3050-bib-0007] Baudinet, J. , Simic, M. , Griffiths, H. , Donnelly, C. , Stewart, C. , & Goddard, E. (2020). Targeting maladaptive overcontrol with radically open dialectical behaviour therapy in a day programme for adolescents with restrictive eating disorders: An uncontrolled case series. Journal of Eating Disorders, 8(1), 68. 10.1186/s40337-020-00338-9 33292696 PMC7663904

[erv3050-bib-0008] Beavers, R. , & Hampson, R. B. (2000). The beavers systems model of family functioning. Journal of Family Therapy, 22(2), 128–143. 10.1111/1467-6427.00143

[erv3050-bib-0009] Beavers, W. , & Hampson, R. (1993). Measuring family competence: The Beavers systems model. In F. Walsh (Ed.), Normal family processes (2nd ed.). Guilford Press.

[erv3050-bib-0010] Byrne, C. E. , Accurso, E. C. , Arnow, K. D. , Lock, J. , & Le Grange, D. (2015). An exploratory examination of patient and parental self‐efficacy as predictors of weight gain in adolescents with anorexia nervosa: Exploratory examination. International Journal of Eating Disorders, 48(7), 883–888. 10.1002/eat.22376 25808269 PMC4845658

[erv3050-bib-0011] Carrot, B. , Duclos, J. , Barry, C. , Radon, L. , Maria, A.‐S. , Kaganski, I. , Jeremic, Z. , Barton‐Clegg, V. , Corcos, M. , Lasfar, M. , Gerardin, P. , Harf, A. , Moro, M.‐R. , Blanchet, C. , & Godart, N. (2019). Multicenter randomized controlled trial on the comparison of multi‐family therapy (MFT) and systemic single‐family therapy (SFT) in young patients with anorexia nervosa: Study protocol of the THERAFAMBEST study. Trials, 20(1), 249. 10.1186/s13063-019-3347-y 31039797 PMC6492384

[erv3050-bib-0012] Dawson, L. , Baudinet, J. , Tay, E. , & Wallis, A. (2018). Creating community—The introduction of multi‐family therapy for eating disorders in Australia. Australian and New Zealand Journal of Family Therapy, 39(3), 283–293. 10.1002/anzf.1324

[erv3050-bib-0013] Dimitropoulos, G. , Freeman, V. E. , Allemang, B. , Couturier, J. , McVey, G. , Lock, J. , & Le Grange, D. (2015). Family‐based treatment with transition age youth with anorexia nervosa: A qualitative summary of application in clinical practice. Journal of Eating Disorders, 3(1), 1. 10.1186/s40337-015-0037-3 25685349 PMC4329223

[erv3050-bib-0014] Dimitropoulos, G. , Herschman, J. , Toulany, A. , & Steinegger, C. (2016). A qualitative study on the challenges associated with accepting familial support from the perspective of transition‐age youth with eating disorders. Eating Disorders, 24(3), 255–270. 10.1080/10640266.2015.1064276 26212112

[erv3050-bib-0015] Dimitropoulos, G. , Landers, A. L. , Msw, V. F. , Ma, J. N. , Garber, A. , & Grange, D. L. (2018). Open trial of family‐based treatment of anorexia nervosa for transition age youth. Journal of the Canadian Academy of Child and Adolescent Psychiatry, 27(1). PMC5777691 29375633

[erv3050-bib-0016] Dodge, L. , Baudinet, J. , Austin, A. , Eisler, I. , Le Grange, D. , & Dimitropoulos, G. Family therapy for emerging adults with anorexia nervosa: Current evidence, practice guidelines, and future directions. European Eating Disorders Review. (in press). 10.1002/erv.3129 PMC12694684 39154324

[erv3050-bib-0017] Doyle, P. M. , Le Grange, D. , Loeb, K. , Doyle, A. C. , & Crosby, R. D. (2010). Early response to family‐based treatment for adolescent anorexia nervosa. International Journal of Eating Disorders, 43(7), 659–662. 10.1002/eat.20764 19816862 PMC8693442

[erv3050-bib-0018] Eisler, I. , Simic, M. , Blessitt, E. , & Dodge, L. , & MCCAED Team . (2016). Maudsley service manual for child and adolescent eating disorders. Retrieved from https://mccaed.slam.nhs.uk/wp‐content/uploads/2019/11/Maudsley‐Service‐Manual‐for‐Child‐and‐Adolescent‐Eating‐Disorders‐July‐2016.pdf

[erv3050-bib-0019] Eisler, I. , Simic, M. , Hodsoll, J. , Asen, E. , Berelowitz, M. , Connan, F. , Ellis, G. , Hugo, P. , Schmidt, U. , Treasure, J. , Yi, I. , & Landau, S. (2016). A pragmatic randomised multi‐centre trial of multifamily and single family therapy for adolescent anorexia nervosa. BMC Psychiatry, 16(1), 422. 10.1186/s12888-016-1129-6 27881106 PMC5122159

[erv3050-bib-0020] Eisler, I. , Simic, M. , Russell, G. F. M. , & Dare, C. (2007). A randomised controlled treatment trial of two forms of family therapy in adolescent anorexia nervosa: A five‐year follow‐up. The Journal of Child Psychology and Psychiatry and Allied Disciplines, 48(6), 552–560. 10.1111/j.1469-7610.2007.01726.x 17537071

[erv3050-bib-0021] Escoffié, A. , Pretorius, N. , & Baudinet, J. (2022). Multi‐family therapy for bulimia nervosa: A qualitative pilot study of adolescent and family members’ experiences. Journal of Eating Disorders, 10(1), 91. 10.1186/s40337-022-00606-w 35786421 PMC9250718

[erv3050-bib-0022] Fairburn, C. G. , & Cooper, Z. (1993). The eating disorder examination. Binge Eating: Nature, Assessment, and Treatment, 317–360.12th ed.

[erv3050-bib-0023] Fink, K. , Rhodes, P. , Miskovic‐Wheatley, J. , Wallis, A. , Touyz, S. , Baudinet, J. , & Madden, S. (2017). Exploring the effects of a family admissions program for adolescents with anorexia nervosa. Journal of Eating Disorders, 5(1), 51. 10.1186/s40337-017-0181-z 29163941 PMC5686912

[erv3050-bib-0024] Fisher, C. A. , Skocic, S. , Rutherford, K. A. , & Hetrick, S. E. (2019). Family therapy approaches for anorexia nervosa. Cochrane Database of Systematic Reviews, 2019(5). 10.1002/14651858.CD004780.pub4 PMC6497182 31041816

[erv3050-bib-0025] Forsberg, S. , LoTempio, E. , Bryson, S. , Fitzpatrick, K. K. , Le Grange, D. , & Lock, J. (2014). Parent‐therapist alliance in family‐based treatment for adolescents with anorexia nervosa: Parent alliance in FBT. European Eating Disorders Review, 22(1), 53–58. 10.1002/erv.2242 23861093

[erv3050-bib-0026] Gorrell, S. , Byrne, C. E. , Trojanowski, P. J. , Fischer, S. , & Le Grange, D. (2022). A scoping review of non‐specific predictors, moderators, and mediators of family‐based treatment for adolescent anorexia and bulimia nervosa: A summary of the current research findings. Eating and Weight Disorders ‐ Studies on Anorexia, Bulimia and Obesity, 27(6), 1971–1990. 10.1007/s40519-022-01367-w PMC9872820 35092554

[erv3050-bib-0027] Graves, T. A. , Tabri, N. , Thompson‐Brenner, H. , Franko, D. L. , Eddy, K. T. , Bourion‐Bedes, S. , Brown, A. , Constantino, M. J. , Flückiger, C. , Forsberg, S. , Isserlin, L. , Couturier, J. , Paulson Karlsson, G. , Mander, J. , Teufel, M. , Mitchell, J. E. , Crosby, R. D. , Prestano, C. , Satir, D. A. , & Thomas, J. J. (2017). A meta‐analysis of the relation between therapeutic alliance and treatment outcome in eating disorders. International Journal of Eating Disorders, 50(4), 323–340. 10.1002/eat.22672 28152196

[erv3050-bib-0028] Hamadi, L. , & Holliday, J. (2020). Moderators and mediators of outcome in treatments for anorexia nervosa and bulimia nervosa in adolescents: A systematic review of randomized controlled trials. International Journal of Eating Disorders, 53(1), 3–19. 10.1002/eat.23159 31506978

[erv3050-bib-0029] Herpertz‐Dahlmann, B. , Borzikowsky, C. , Altdorf, S. , Heider, K. , Dempfle, A. , & Dahmen, B. (2021). ‘Therapists in action’—Home treatment in adolescent anorexia nervosa: A stepped care approach to shorten inpatient treatment. European Eating Disorders Review, 29(3), 427–442. 10.1002/erv.2755 32558214

[erv3050-bib-0030] Hilbert, A. , Hoek, H. W. , & Schmidt, R. (2017). Evidence‐based clinical guidelines for eating disorders: International comparison. Current Opinion in Psychiatry, 30(6), 423–437. 10.1097/YCO.0000000000000360 28777107 PMC5690314

[erv3050-bib-0031] Hilton, C. , Jones, S. , Akers, N. , Panagaki, K. , & Sellwood, W. (2022). Self‐report measures assessing aspects of personal recovery in relatives and other informal carers of those with psychosis: A systematic review. Frontiers in Psychology, 13, 926981. 10.3389/fpsyg.2022.926981 35911034 PMC9335122

[erv3050-bib-0032] Hoste, R. R. (2015). Incorporating family‐based therapy principles into a partial hospitalization programme for adolescents with anorexia nervosa: Challenges and considerations: FBT principles for anorexia nervosa. Journal of Family Therapy, 37(1), 41–60. 10.1111/1467-6427.12055

[erv3050-bib-0033] Jewell, T. , Blessitt, E. , Stewart, C. , Simic, M. , & Eisler, I. (2016). Family therapy for child and adolescent eating disorders: A critical review. Family Process, 55(3), 577–594. 10.1111/famp.12242 27543373

[erv3050-bib-0034] Jewell, T. , Herle, M. , Serpell, L. , Eivors, A. , Simic, M. , Fonagy, P. , & Eisler, I. (2021). Attachment and mentalization as predictors of outcome in family therapy for adolescent anorexia nervosa. European Child & Adolescent Psychiatry, 32(7), 1241–1251. 10.1007/s00787-021-01930-3 34967934 PMC10276078

[erv3050-bib-0035] Kinston, W. , & Loader, P. (1984). Eliciting whole‐family interaction with a standardized clinical interview. Journal of Family Therapy, 6(3), 347–363. 10.1046/j.1467-6427.1984.00655.x

[erv3050-bib-0036] Le Grange, D. , Accurso, E. C. , Lock, J. , Agras, S. , & Bryson, S. W. (2014). Early weight gain predicts outcome in two treatments for adolescent anorexia nervosa: Early Weight Gain for Adolescent Anorexia Nervosa. International Journal of Eating Disorders, 47(2), 124–129. 10.1002/eat.22221 24190844 PMC4341963

[erv3050-bib-0037] Le Grange, D. , Hoste, R. R. , Lock, J. , & Bryson, S. W. (2011). Parental expressed emotion of adolescents with anorexia nervosa: Outcome in family‐based treatment. International Journal of Eating Disorders, 44(8), 731–734. 10.1002/eat.20877 22072411 PMC3117016

[erv3050-bib-0038] Le Grange, D. , Hughes, E. K. , Court, A. , Yeo, M. , Crosby, R. D. , & Sawyer, S. M. (2016). Randomized clinical trial of parent‐focused treatment and family‐based treatment for adolescent anorexia nervosa. Journal of the American Academy of Child & Adolescent Psychiatry, 55(8), 683–692. 10.1016/j.jaac.2016.05.007 27453082

[erv3050-bib-0039] Le Grange, D. , Lock, J. , Agras, W. S. , Moye, A. , Bryson, S. W. , Jo, B. , & Kraemer, H. C. (2012). Moderators and mediators of remission in family‐based treatment and adolescent focused therapy for anorexia nervosa. Behaviour Research and Therapy, 50(2), 85–92. 10.1016/j.brat.2011.11.003 22172564 PMC3260378

[erv3050-bib-0040] Madden, S. , Miskovic‐Wheatley, J. , Wallis, A. , Kohn, M. , Lock, J. , Le Grange, D. , Jo, B. , Clarke, S. , Rhodes, P. , Hay, P. , & Touyz, S. (2015). A randomized controlled trial of in‐patient treatment for anorexia nervosa in medically unstable adolescents. Psychological Medicine, 45(2), 415–427. 10.1017/S0033291714001573 25017941 PMC4301212

[erv3050-bib-0041] McClelland, J. , Simic, M. , Schmidt, U. , Koskina, A. , & Stewart, C. (2020). Defining and predicting service utilisation in young adulthood following childhood treatment of an eating disorder. BJPsych Open, 6(3), e37. 10.1192/bjo.2020.13 32248870 PMC7176893

[erv3050-bib-0042] McRae, K. (2016). Cognitive emotion regulation: A review of theory and scientific findings. Current Opinion in Behavioral Sciences, 10, 119–124. 10.1016/j.cobeha.2016.06.004

[erv3050-bib-0043] Mokkink, L. B. , Terwee, C. B. , Patrick, D. L. , Alonso, J. , Stratford, P. W. , Knol, D. L. , Bouter, L. M. , & de Vet, H. C. W. (2010). The COSMIN checklist for assessing the methodological quality of studies on measurement properties of health status measurement instruments: An international Delphi study. Quality of Life Research, 19(4), 539–549. 10.1007/s11136-010-9606-8 20169472 PMC2852520

[erv3050-bib-0044] Monteleone, A. M. , Pellegrino, F. , Croatto, G. , Carfagno, M. , Hilbert, A. , Treasure, J. , Wade, T. , Bulik, C. M. , Zipfel, S. , Hay, P. , Schmidt, U. , Castellini, G. , Favaro, A. , Fernandez‐Aranda, F. , Il Shin, J. , Voderholzer, U. , Ricca, V. , Moretti, D. , Busatta, D. , & Solmi, M. (2022). Treatment of eating disorders: A systematic meta‐review of meta‐analyses and network meta‐analyses. Neuroscience & Biobehavioral Reviews, 142, 104857. 10.1016/j.neubiorev.2022.104857 36084848 PMC9813802

[erv3050-bib-0045] Nyman‐Carlsson, E. , Norring, C. , Engström, I. , Gustafsson, S. A. , Lindberg, K. , Paulson‐Karlsson, G. , & Nevonen, L. (2020). Individual cognitive behavioral therapy and combined family/individual therapy for young adults with anorexia nervosa: A randomized controlled trial. Psychotherapy Research, 30(8), 1011–1025. 10.1080/10503307.2019.1686190 31709920

[erv3050-bib-0046] Potterton, R. , Richards, K. , Allen, K. , & Schmidt, U. (2020). Eating disorders during emerging adulthood: A systematic scoping review. Frontiers in Psychology, 10, 3062. 10.3389/fpsyg.2019.03062 32082210 PMC7005676

[erv3050-bib-0047] Richards, I. L. , Subar, A. , Touyz, S. , & Rhodes, P. (2018). Augmentative approaches in family‐based treatment for adolescents with restrictive eating disorders: A systematic review. European Eating Disorders Review, 26(2), 92–111. 10.1002/erv.2577 29282801

[erv3050-bib-0048] Rockwell, R. E. , Boutelle, K. , Trunko, M. E. , Jacobs, M. J. , & Kaye, W. H. (2011). An innovative short‐term, intensive, family‐based treatment for adolescent anorexia nervosa: Case series: Anorexia nervosa family therapy. European Eating Disorders Review, 19(4), 362–367. 10.1002/erv.1094 21308869

[erv3050-bib-0049] Rose, J. S. , Vaewsorn, A. , Rosselli‐Navarra, F. , Wilson, G. T. , & Weissman, R. S. (2013). Test‐retest reliability of the eating disorder examination‐questionnaire (EDE‐Q) in a college sample. Journal of Eating Disorders, 1(1), 42. 10.1186/2050-2974-1-42 24999420 PMC4081765

[erv3050-bib-0050] Simic, M. , Baudinet, J. , Blessitt, E. , Wallis, A. , & Eisler, I. (2021). Multi‐family therapy for anorexia nervosa: A treatment manual (1st ed.). Routledge. 10.4324/9781003038764

[erv3050-bib-0051] Simic, M. , & Eisler, I. (2015). Multi‐family therapy. In K. L. Loeb , D. Le Grange , & J. Lock (Eds.), Family therapy for adolescent eating and weight disorders (1st ed., pp. 110–138). Imprint Routledge.

[erv3050-bib-0052] Simic, M. , Jewell, T. , & Eisler, I. (2020). Beneath the surface of expressed emotion: The clinical relevance of possible mechanisms underlying EE in eating disorder. In R. R. Hoste & D. Le Grange (Eds.), Expressed emotion and eating disorders. Guilford Press.

[erv3050-bib-0053] Simic, M. , Stewart, C. S. , Eisler, I. , Baudinet, J. , Hunt, K. , O’Brien, J. , & McDermott, B. (2018). Intensive treatment program (ITP): A case series service evaluation of the effectiveness of day patient treatment for adolescents with a restrictive eating disorder. International Journal of Eating Disorders, 51(11), 1261–1269. 10.1002/eat.22959 30265750

[erv3050-bib-0054] StataCorp (2021). Stata statistical software: Release 17. StataCorp LP. [Computer software].

[erv3050-bib-0055] Stewart, C. S. , Baudinet, J. , Hall, R. , Fiskå, M. , Pretorius, N. , Voulgari, S. , Hunt, K. , Eisler, I. , & Simic, M. (2019). Multi‐family therapy for bulimia nervosa in adolescence: A pilot study in a community eating disorder service. Eating Disorders: The Journal of Treatment & Prevention, 29(4), 351–367. Social Science Premium Collection. 10.1080/10640266.2019.1656461 31609163

[erv3050-bib-0056] Szmukler, G. I. , Burgess, P. , Herrman, H. , Bloch, S. , Benson, A. , & Colusa, S. (1996). Caring for relatives with serious mental illness: The development of the Experience of Caregiving Inventory. Social Psychiatry and Psychiatric Epidemiology, 31(3–4), 137–148. 10.1007/BF00785760 8766459

[erv3050-bib-0057] Tantillo, M. , McGraw, J. S. , & Le Grange, D. (2020). Multifamily therapy group for young adults with anorexia nervosa: Reconnecting for recovery (1st ed.). Routledge. 10.1002/eat.23097 31150141

[erv3050-bib-0058] Treasure, J. , Schmidt, U. , & Hugo, P. (2005). Mind the gap: Service transition and interface problems for patients with eating disorders. British Journal of Psychiatry, 187(5), 398–400. 10.1192/bjp.187.5.398 16260812

[erv3050-bib-0059] Vall, E. , & Wade, T. D. (2015). Predictors of treatment outcome in individuals with eating disorders: A systematic review and meta‐analysis: Predictors of treatment outcomes in individuals with eating disorders. International Journal of Eating Disorders, 48(7), 946–971. 10.1002/eat.22411 26171853

[erv3050-bib-0065] Wickham, H. (2016). ggplot2: Elegant graphics for data analysis. ISBN 978‐3‐319‐24277‐4. Springer‐Verlag New York. https://ggplot2.tidyverse.org

[erv3050-bib-0060] Wiseman, H. , Ensoll, S. , Russouw, L. , & Butler, C. (2019). Multi‐family therapy for young people with anorexia nervosa: Clinicians’ and carers’ perspectives on systemic changes. Journal of Systemic Therapies, 38(3), 67–83. 10.1521/jsyt.2019.38.3.67

